# Growth Arrest-Specific 6 Enhances the Suppressive Function of CD4^+^CD25^+^ Regulatory T Cells Mainly through Axl Receptor

**DOI:** 10.1155/2017/6848430

**Published:** 2017-02-08

**Authors:** Guang-ju Zhao, Jia-yi Zheng, Jia-lan Bian, Long-wang Chen, Ning Dong, Yan Yu, Guang-liang Hong, Arvine Chandoo, Yong-Ming Yao, Zhong-qiu Lu

**Affiliations:** ^1^Emergency Department, The First Affiliated Hospital of Wenzhou Medical University, Wenzhou 325000, China; ^2^Burns Institute, First Hospital Affiliated to the Chinese PLA General Hospital, Beijing 100048, China; ^3^Department of Gastrointestinal Surgery, The First Affiliated Hospital of Wenzhou Medical University, Wenzhou 325000, China

## Abstract

*Background.* Growth arrest-specific (Gas) 6 is one of the endogenous ligands of TAM receptors (Tyro3, Axl, and Mertk), and its role as an immune modulator has been recently emphasized. Naturally occurring CD4^+^CD25^+^ regulatory T cells (Tregs) are essential for the active suppression of autoimmunity. The present study was designed to investigate whether Tregs express TAM receptors and the potential role of Gas6-TAM signal in regulating the suppressive function of Tregs.* Methods.* The protein and mRNA levels of TAM receptors were determined by using Western blot, immunofluorescence, flow cytometry, and RT-PCR. Then, TAM receptors were silenced using targeted siRNA or blocked with specific antibody. The suppressive function of Tregs was assessed by using a CFSE-based T cell proliferation assay. Flow cytometry was used to determine the expression of Foxp3 and CTLA4 whereas cytokines secretion levels were measured by ELISA assay.* Results.* Tregs express both Axl and Mertk receptors. Gas6 increases the suppressive function of Tregs in vitro and in mice. Both Foxp3 and CTLA-4 expression on Tregs are enhanced after Gas6 stimulation. Gas6 enhances the suppressive activity of Tregs mainly through Axl receptor.* Conclusion*. Gas6 has a direct effect on the functions of CD4^+^CD25^+^Tregs mainly through its interaction with Axl receptor.

## 1. Introduction

As inhibitors of immune responses and inflammation, naturally occurring CD4^+^CD25^+^Tregs play a crucial role in controlling the extent of cell-mediated immunity and preventing excessive immune-induced organ damage [1, 2]. On the other hand, the enhanced suppressive function of Tregs may create an immunosuppressive state [1, 2]. Recently, dysregulation of Tregs has been observed in various diseases, including tumor, rheumatoid arthritis, diabetes, and infection diseases [3–6]. In this view, Treg cell-based therapies have held great promise for restoring the equilibrium of immune response [7, 8]; it is important to understand the mechanism of function changes of those cells.

Growth arrest-specific (Gas) 6 structurally belongs to the family of plasma vitamin K-dependent proteins and has growth factor-like properties through its interaction with receptor tyrosine kinases of the TAM family; Tyro3, Axl, and Mertk [9, 10]. Activation of Axl by Gas6 can induce chemotaxis of dendritic cells (DCs) which can prevent them from undergoing apoptosis [11]. Gas6 can further decrease cytokine secretion by lipopolysaccharides (LPS) stimulated monocytes/macrophages [12]. Additionally, Mertk-deficient mice are hypersensitive to LPS-induced endotoxic shock, tissue damage, and death due to the excessive production of TNF-*α* [13]. Thus, TAM receptors have been recognized as a brake for the innate immunity.

Although the role of Gas6/TAM signaling in innate immunity has been well documented, the effect and mechanism of it in adaptive immunity remain largely elusive. Previous studies illustrated that mice lacking all three TAM receptors develop a severe lymphoproliferative disorder accompanied by broad-spectrum autoimmunity [14]. Because TAM receptors are not expressed in most lymphocytes, the lymphoproliferative disorder in TAM receptors knockout mice was attributed to hyperactivation of antigen-presenting cells [14–16]. However, in the present study, we find that Gas6 increases the suppressive effect of CD4^+^CD25^+^Tregs on effector CD4^+^T cells. We also define the Treg cell as a direct target of Gas6. CD4^+^CD25^+^Tregs express Axl and Mertk receptors, and Gas6 enhances the suppressive capacity of CD4^+^CD25^+^Tregs mainly through Axl receptor. Our results provide new insights into the functions of Gas6/Axl signaling in immune responses and inflammation diseases.

## 2. Materials and Methods

### 2.1. Mice

Male BALB/c mice (6–8 weeks, purchased from the Laboratory Animal Institute, Chinese Academy of Medical Sciences, Beijing, China) were housed in separate cages in a temperature-controlled room with 12 h light and 12 h darkness and allowed to acclimatize for at least 7 days before being used. All methods were carried out in accordance with the National Institute of Health Guide for the care and use of Laboratory Animal, and all experimental protocols were approved by the ethics committee of the Laboratory Animal Ethics Committee of Wenzhou Medical University & Laboratory Animal Centre of Wenzhou Medical University.

### 2.2. rmGas6 Administration

To investigate the effect of Gas6 on CD4^+^CD25^+^Tregs in vivo, healthy mice were administered 1, 3, or 6 *μ*g/mouse of rmGas6 (8310-GS, R&D Systems, Minneapolis, MN) via tail vein. Control animals received the same volume of normal saline.

### 2.3. Cell Preparation and Culture

CD4^+^CD25^+^Tregs and CD4^+^CD25^−^T cells were isolated from murine splenocytes by magnetic cell sorting (Miltenyi Biotec, Bergisch Gladbach, Germany), according to the manufacturer's instructions, as described previously [17, 18]. The purity of isolated CD4^+^CD25^+^Tregs and CD4^+^CD25^−^T cells was determined at 92% to 95% by flow cytometric analysis (see Fig. S1 in Supplementary Material available online at https://doi.org/10.1155/2017/6848430).

CD4^+^CD25^+^Tregs cultures were set up at a density of 10^6^ cells/ml in complete RPMI 1640 medium supplemented with 10% fetal calf serum, 100 U/ml penicillin, 100 *μ*g/ml streptomycin, and 50 *μ*M 2-Mercaptoethanol. Gas6, in different concentration, was used to stimulate Tregs. Previously studies illustrated that the antibodies raised against the extracellular N-terminus domains of the TAM receptors could block the function of the respective TAM receptor family members [19–21]. In some experiments, CD4^+^CD25^+^Tregs were pretreated with 30 *μ*g/ml anti-Axl antibody (clone 107332) or 30 *μ*g/ml anti-Mertk antibody (clone 108921) (R&D Systems, Minneapolis, MN) 1 hour before stimulation with Gas6.

### 2.4. Lentiviral Vector Production and Cell Transduction

The siRNA-expression lentivirus vectors were constructed, packed, and purified by Bioyong Technologies Inc. (Beijing, China). The sequences used for Axl knockdown were sense, 5′-GAGAUGGACAGAUCCUAGA-3′, and antisense, 5′-UCUAGGAUCUGUCCAUCUC-3′. The sequences for control siRNA were sense, 5′-CCUACGCCACCAAUUUCGU-3′, and antisense, 5′-ACGAAAUUGGUGGCG-UAGG-3′, as described previously [21]. CD4^+^CD25^+^T cells were resuspended to 10^6^/ml in 4 ml of complete medium (RPMI 1640 with 10% fetal bovine serum) in 25 cm^2^ dishes. After being cultured for 12 h, cells were washed, counted, and plated at 5 × 10^5^ in complete medium in 25 cm^2^ dishes. Lentivirus was added to a final multiplicity of infection (MOI) of 30 colony-forming units (CFU) per cell. In initial experiments, polybrene was added to give a final concentration of 5 mg/ml. After 16 h, the cells were washed with PBS and resuspended to 10^6^/ml in fresh medium.

### 2.5. Cell Viability Assay

The viability of CD4^+^CD25^+^Tregs cells was determined by CCK-8 according to protocols provided by the manufacturer (Dojindo Laboratories, Kumamoto, Japan). The absorbance was read in microplate reader (Spectra MR, Dynex, Richfield, MN) at OD450 nm.

### 2.6. Western Blot

Western blot was performed to determine the expression of TAM receptors in mouse CD4^+^CD25^+^Tregs and CD4^+^CD25^−^T cells. In some experiments, cardiac tissue was used as positive control for Axl, and kidney tissue was used as positive control for Tyro3 and Mertk. Cell and tissue lysis, SDS-PAEG separation, transfer to nitrocellulose membranes, and development of blots using an ECL system (Santa Cruz Biotechnology, Santa Cruz, CA) were performed as described previously. Membranes were probed with following antibodies: rat anti-Axl (clone 107332), anti-Tyro3 (clone 109646), and anti-Mertk (clone 108921) antibodies were obtained from R&D systems (Minneapolis, MN). Rabbit anti-*β*-actin antibody was purchased from BD Biosciences (Mountain View, CA). The protein levels of TAM receptors were quantified by densitometry using NIH image software and normalized to *β*-actin levels.

### 2.7. Semiquantitative Reverse Transcription-Polymerase Chain Reaction Analysis

Total RNA was prepared from freshly isolated CD4^+^CD25^+^Tregs and macrophages (positive control) using TRIzol reagent (Invitrogen, Carlsbad, CA). The cDNA was prepared from 2 *μ*g of RNA using Promega PCR Master mix (Promega, Madison, WI). Aliquots (4 *μ*l) of cDNA were amplified by PCR using Supermix in a thermal cycler (Applied Biosystems, Foster City, CA) with specific primers. The gene-specific primer sets for Axl receptor, Mertk receptor, and *β*-actin were purchased from SBS Genetech (Beijing, China). The primers for mouse Axl receptor were 5′-TGAGCCAACCCGTGGAAAGAG-3′ (forward) and 5′-GAAAACAGACGCCAATGAGCC-3′ (reverse). The primers for mouse Mertk receptor were 5′-AAGGTAGATTACGCACCCTCGTC-3′ (forward) and 5′-GAAACTCCTCGATCTCCCGTTG-3′ (reverse). For *β*-actin, we used the following primer: 5′-GAGACCTTCAACACCCCAGC-3′ (forward) and 5′-reverse CCACAGGATTCCATACCCAA-3′ (reverse). A sample containing all reaction reagents except cDNA was used as PCR negative control in each experiment. The absence of genomic DNA was verified using RNA that was reverse-transcribed without the enzyme (RT−). The cDNA was amplified by 40 PCR cycles (denaturation at 94°C for 3 min, annealing at 59°C for 30 s, and extension at 72°C for 1 min, with the final extension of one cycle at 72°C for 5 min). Amplified fragments of expected size were analyzed using a 2% agarose gel and were photographed under UV light.

### 2.8. Immunofluorescence

Freshly isolated CD4^+^CD25^+^Tregs (2 × 10^5^) were washed with PBS for three times and fixed with 4% paraformaldehyde in PBS at room temperature. Cells were blocked with 1% bovine serum albumin in PBS for 30 min. Then, cells were incubated with 20 *μ*g/ml anti-Axl (clone 175128) (R&D Systems, Minneapolis, MN) or 20 *μ*g/ml anti-Mertk (clone 108921) (R&D Systems, Minneapolis, MN) monoclonal Abs overnight at 4°C. After washing with PBS, Tregs were incubated with FITC-labeled goat anti-rat IgG (1 : 30, sigma) as the secondary Ab for 30 min at room temperature followed by 3x PBS washes. Images were observed with aEclipse Ti inverted epifluorescence microscope (Nikon, Tokyo, Japan) and a laser scanning confocal microscope (Leica, Mannheim, Germany).

### 2.9. Flow Cytometric Analysis

To detect the expression of CTLA4 and Foxp3, CD4^+^CD25^+^Tregs (2 × 10^5^) were stained with hamster anti-mouse CTLA4-FITC antibody and an isotype (2 *μ*g/ml, clone 1B8) (Southern Biotechnology Associates Inc., Birmingham, AL) or anti-mouse/rat Foxp3 PerCP-Cyanine5.5 antibody and an isotype (2 *μ*g/ml, clone FJK-16s) (eBioscience, San Diego, CA), respectively. To detect the expression of Axl and Mertk receptors, freshly isolated CD4^+^CD25^+^Tregs (2 × 10^5^) were washed with PBS for three times and then incubated with anti-Axl antibody and an isotype (clone 175128) (R&D Systems, Minneapolis, MN) or anti-Mertk antibody and an isotype (clone 108921) (R&D Systems, Minneapolis, MN) monoclonal Ab for 30 min in FACS buffer (PBS containing 2% bovine serum albumin and 0.1% sodium azide) at 4°C in the dark. After washing with PBS, Tregs were incubated with FITC-labeled goat anti-rat IgG as the secondary Ab for 15 min at room temperature. All cells were analyzed by flow cytometry using a FACScan (BD Biosciences, Mountain View, CA).

### 2.10. In Vitro Suppression Assays

The normal CD4^+^CD25^−^T cells were labeled with carboxyfluorescein-succinimidyl-ester (CFSE, Invitrogen, Carlsbad, CA) according to the manufacturer's instructions and then mixed with CD4^+^CD25^+^Tregs from rmGas6-treated or normal mice in a ratio of 1 : 1. Anti-CD3 monoclonal antibody (clone 145-2C11) (mAb) combined with anti-CD28 mAb (clone 37.51) (eBioscience, San Diego, CA) at a final concentration of 1 *μ*g/ml was added to the culture for stimulation. The proliferation of cells was measured by flow cytometry using a FACScan (BD Biosciences, Mountain View, CA). The percentage of suppression was calculated as [1 − (% Teff proliferation with Treg cells/% Teff proliferation without Treg cells)] × 100.

### 2.11. Enzyme-Linked Immunosorbent Assays

IL-10 and TGF-*β*1 levels in Treg culture supernatants were measured using ELISA kits obtained from Genetimes Technology Inc. (Shanghai, China), according to the manufacturer's instructions. To assess T cell cytokine production, culture supernatants from CD4^+^CD25^+^Tregs and CD4^+^CD25^−^Tcells cocultures were collected, and IL-2, IL-4, and IFN-*γ* amounts were measured using ELISA kits purchased from Genetimes Technology Inc. (Shanghai, China).

### 2.12. Statistical Analysis

Data were represented as mean ± standard deviation (SD) and were analyzed with a one-way ANOVA. Fisher's least significant difference (LSD) was used to evaluate significant differences between groups. *P* values < 0.05 were considered statistically significant.

## 3. Results

### 3.1. TAM Receptors Expression on CD4^+^CD25^+^Tregs and CD4^+^CD25^−^T Cells

**Figure 1:**
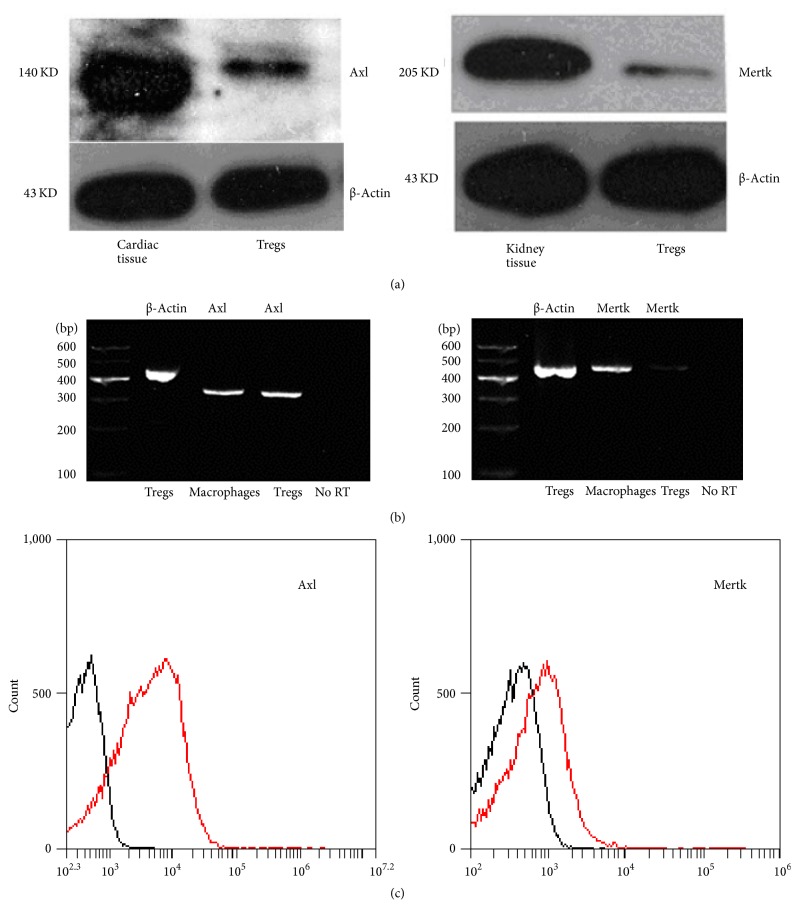
TAM receptors were expressed in mouse CD4^+^CD25^+^Tregs. (a) Protein expression from CD4^+^CD25^+^Tregs, cardiac tissue, and kidney tissue was analyzed by Western blot using specific anti-Axl (2 *μ*g/ml) and anti-Mertk (2 *μ*g/ml) antibodies. (b) Axl and Mertk mRNA expressions in CD4^+^CD25^+^Tregs and marcrophages were determined by semiquantitative RT-PCR. Expression of *β*-actin was shown as an internal control. (c) CD4^+^CD25^+^Tregs were incubated with anti-Axl (10 *μ*g/ml) and anti-Mertk (10 *μ*g/ml) monoclonal Ab, respectively, followed by FITC-labeled goat anti-rat IgG as the secondary Ab (1 : 30). The cells were analyzed by flow cytometry.

**Figure 2:**
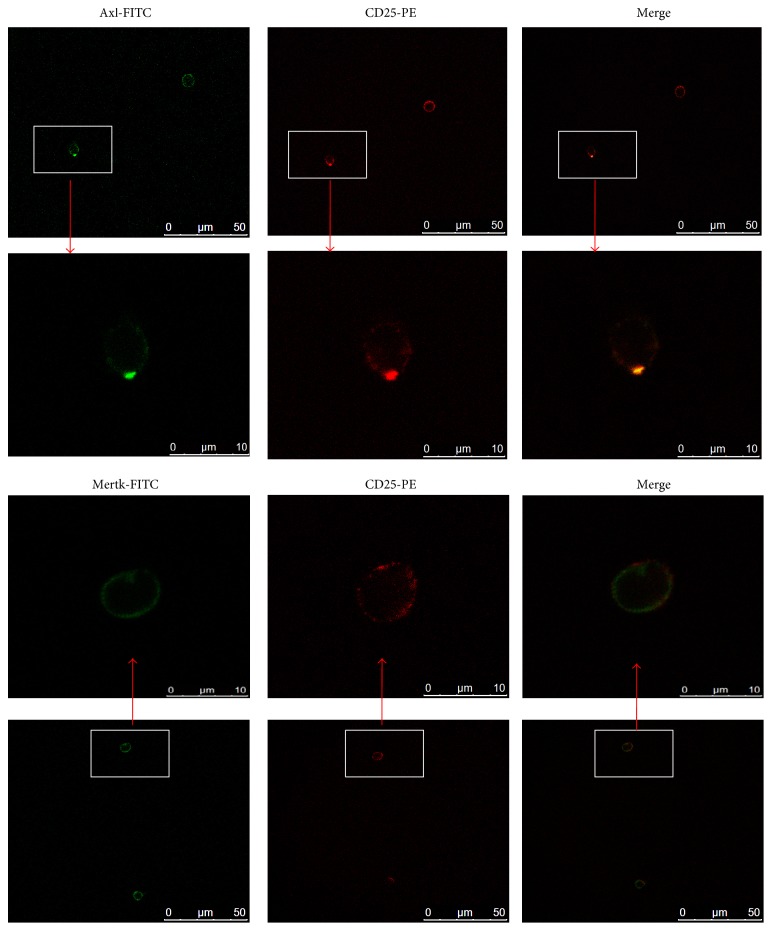
Immunofluorescence detection of TAM receptors. CD4^+^CD25^+^Tregs were incubated with anti-Axl (10 *μ*g/ml) and anti-Mertk (10 *μ*g/ml) monoclonal Ab, respectively, followed by FITC-labeled goat anti-rat IgG as the secondary Ab (1 : 30). The cells were analyzed by fluorescent confocal microscopy. Representative photomicrographs show that FITC-positive cells (green) were detected among PE-positive CD4^+^CD25^+^Tregs (red). Those double-stained cells are shown in yellow.

The expression of TAM receptors (Tyro3, Axl, and Mertk) in mouse CD4^+^CD25^+^Tregs was determined by Western blot. Cardiac tissue was used as positive control for Axl, and kidney tissue was used as positive control for Tyro3 and Mertk. We found that CD4^+^CD25^+^Tregs express Axl and Mertk receptors (Figure 1(a)) but do not express Tyro3 receptor (data not shown). Additionally, CD4^+^CD25^−^T cells do not express Axl and Mertk receptors (Fig. S1). The expression of Axl and Mertk receptors at mRNA levels were also detected by RT-PCR, and the mRNA of the two receptors in macrophages were analyzed as positive controls (Figure 1(b)). To further confirm the expressions of the receptors, CD4^+^CD25^+^Tregs were incubated with rat anti-mouse Axl Ab and rat anti-mouse Mertk Ab followed by FITC-labeled goat anti-rat IgG. FITC fluorescence was detected on the cell surface by flow cytometry (Figure 1(c)) and fluorescent confocal microscopy (Figure 2) as well as fluorescence microscopy (Fig. S2).

### 3.2. CD4^+^CD25^+^Tregs from rmGas6-Treated Mice Exhibited Increased Foxp3 Levels and Suppressive Activity

**Figure 3:**
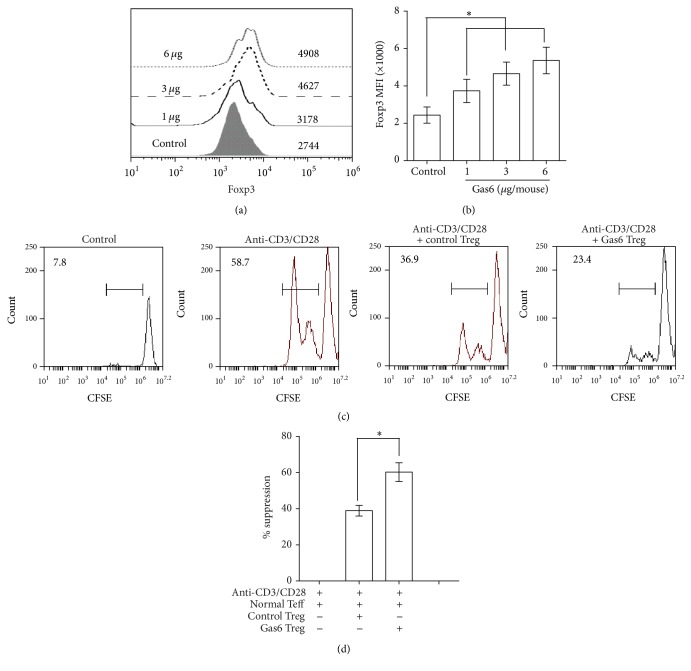
CD4^+^CD25^+^Tregs from rmGas6-treated mice exhibited increased Foxp3 levels and suppressive activity. BALB/c mice were administrated with 1, 3, or 6 *μ*g/mouse of rmGas6 via tail vein. After 24 hours, CD4^+^CD25^+^T cells were purified from spleens. (a-b) MFI analyses showing expression levels of Foxp3 protein in CD4^+^CD25^+^T cells from rmGas6-treated mice and control mice (two mice each in two independent experiments). ^*∗*^*P* < 0.05 compared with the value for the control group. (c) CD4^+^CD25^−^T cells from normal mice were cocultured with CD4^+^CD25^+^T cells from control or Gas6-treated mice for 3 days with or without anti-CD3 and anti-CD28 stimulation (three mice each in two independent experiments). Histogram of CFSE-labeled CD4^+^CD25^−^T cells are displayed, and the percentage of cells that have proliferated as shown in gates. One representative example is shown. (d) Mean percentage of suppression was calculated (*n* = 6). CD4^+^CD25^+^T cells from Gas6-treated mice had increased suppressive activity when compared with those from control mice. ^*∗*^*P* < 0.05 compared with the value for anti-CD3/CD28 group.

To assess the effects of Gas6 on Tregs in vivo, these cells were analyzed at 24 hours in normal animals after different dosage of rmGas6 administration. As shown in Figures 3(a) and 3(b), the MFIs of Foxp3 in CD4^+^CD25^+^Tregs from rmGas6 treated mice were significantly higher compared with those in the Tregs of control mice. In addition, CD4^+^CD25^−^T cells from normal mice were cocultured for 3 days with CD4^+^CD25^+^Tregs from mice treated with rmGas6 or not. The suppressive activity of CD4^+^CD25^+^Tregs was determined by CFSE assay. As shown in Figures 3(c) and 3(d), CD4^+^CD25^+^Tregs from rmGas6 (3 *μ*g/mouse) treated mice exhibited significantly greater suppressive activity compared with the cells from control mice.

### 3.3. Effects of Gas6 on CTLA-4 and Foxp3 Expression in CD4^+^CD25^+^Tregs In Vitro

**Figure 4:**
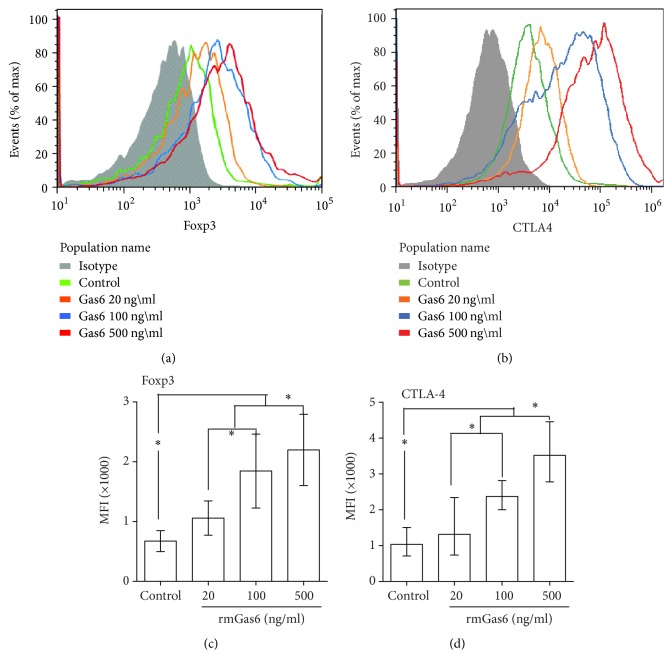
Effects of Gas6 on Foxp3 and CTLA-4 expression in CD4^+^CD25^+^Tregs in vitro. Representative examples of Foxp3 (a) and CTLA-4 (b) expressions in CD4^+^CD25^+^Tregs after 20, 100, and 500 ng/ml rmGas6 stimulation for 24 hours. The average levels of Foxp3 (c) and CTLA-4 (d) were increased after Gas6 stimulation (*n* = 4/group). ^*∗*^*P* < 0.05.

The levels of CTLA-4 and Foxp3 on CD4^+^CD25^+^Tregs after rmGas6 stimulation were determined by flow cytometry. As shown in Figures 3(a) and 3(c), stimulation with various doses of rmGas6 significantly increased Foxp3 expression in CD4^+^CD25^+^Tregs at 24 h. In addition, Tregs stimulated with rmGas6 at 100 ng/ml and 500 ng/ml exhibited an increased expression of CTLA-4 when compared with control cells, while 20 ng/ml rmGas6 did not affect CTLA-4 expression (Figures 4(b) and 4(d)).

### 3.4. Effects of Gas6 on TGF-*β*1 and IL-10 Production in CD4^+^CD25^+^Tregs In Vitro

Upon rmGas6 stimulation, CD4^+^CD25^+^Tregs failed to produce detectable IL-10 in vitro (data not shown). Gas6 in concentrations ranging from 20 ng/ml to 500 ng/ml had no effect on TGF-*β*1 production in CD4^+^CD25^+^Tregs (Fig. S3).

### 3.5. Gas6 Enhances the Suppressive Activity of Tregs In Vitro

**Figure 5:**
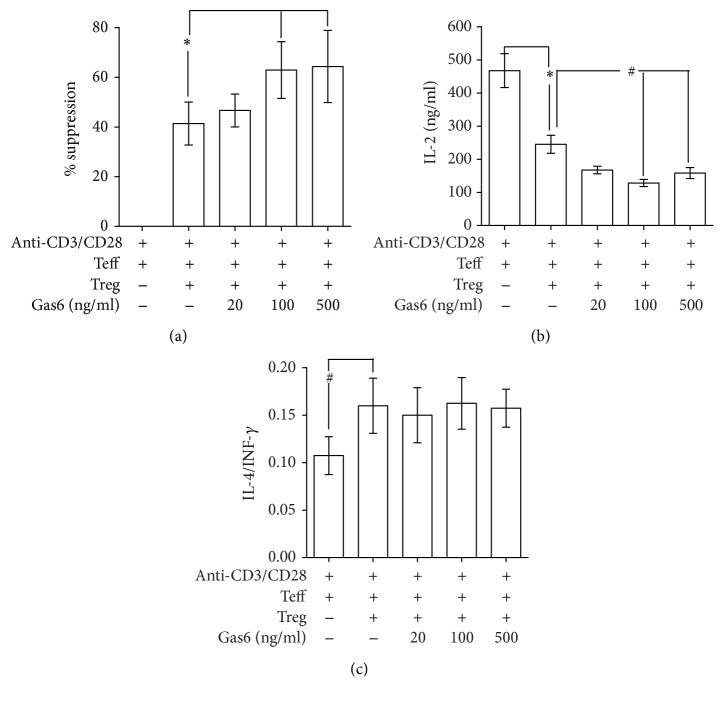
Effects of Gas6 on suppressive activity of CD4^+^CD25^+^Tregs in vitro. CD4^+^CD25^+^Tregs were pretreated with PBS or rmGas6, respectively, for 24 h. Then, prestimualted CD4^+^CD25^+^Tregs were mixed with CD4^+^CD25^−^T cells in the presence of anti-CD3/CD28 Abs. After 72 h, the proliferation of CD4^+^CD25^−^T cells and the levels of cytokine in the conditioned media were determined. (a) Suppressive activity of CD4^+^CD25^+^Tregs was measured by a CFSE assay (*n* = 4/group). The levels of IL-2 (b) as well as IL-4 and IFN-*γ* (c) in the conditioned media were determined by ELISA (*n* = 4/group). ^*∗*^*P* < 0.05 compared with the value for the control Tregs group, and ^#^*P* < 0.05 compared with the value for anti-CD3/CD28 group.

To investigate the effect of Gas6 on the suppressive activity of CD4^+^CD25^+^T cells in vitro, CD4^+^CD25^+^T cells pretreated with or without rmGas6 were mixed with CD4^+^CD25^−^T cells. As shown in Figure 5(a), anti-CD3/CD28 mAbs induced marked proliferation of CD4^+^CD25^−^T cells. After being cocultured with CD4^+^CD25^+^Tregs, the proliferative activity of CD4^+^CD25^−^T cells was significantly decreased. The suppressive function of CD4^+^CD25^+^Tregs was increased after pretreatment with 100 ng/ml and 500 ng/ml rmGas6. Consistent suppression of IL-2 was observed in CD4^+^CD25^+^Treg groups (Figure 5(b)). Gas6 stimulation at 100 ng/ml and 500 ng/ml enhanced such inhibitory response (Figure 5(b)). However, Gas6 stimulation had no effect on CD4^+^CD25^+^Treg-induced Th2 polarization of T cells (Figure 5(c)).

### 3.6. Gas6 Modulates the Suppressive Function of Treg Cells Mainly through Axl Receptor

**Figure 6:**
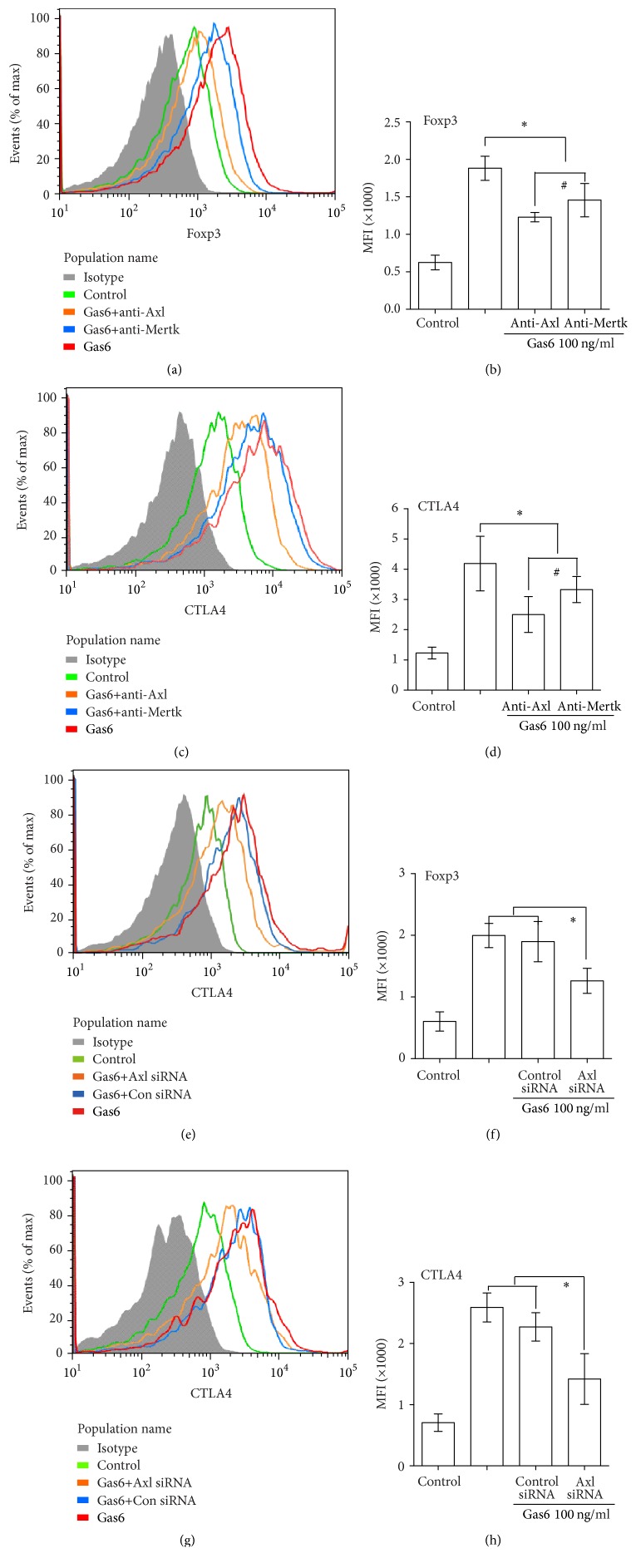
Gas6 modulates the Foxp3 and CTLA4 expression mainly through Axl receptor. (a–d) CD4^+^CD25^+^Tregs were treated with anti-Axl or anti-Mertk Abs or PBS in the presence of 100 ng/ml rmGas6. After 24 h of incubation, the expression of CTLA-4 and Foxp3 was determined by flow cytometry (*n* = 4/group). ^*∗*^*P* < 0.05 compared with the value for the Gas6 group, and ^#^*P* < 0.05 compared with the value for rmGas6+anti-Mertk group. (e–h) The expression of CTLA-4 and Foxp3 in Tregs with or without Axl knockout was determined by flow cytometry. ^*∗*^*P* < 0.05 compared with the value for the Gas6 group.

To investigate the receptor mechanism of Gas6 on the functions of Treg cells, antibodies were used to block the Axl and Mertk receptors, respectively. As shown in Figures 6(a)–6(d), Gas6-induced enhancement of CTLA-4 and Foxp3 expression in Tregs was suppressed by preincubation with anti-Axl Ab and anti-Mertk Ab. Additionally, the suppressive effect of anti-Axl Ab on Gas6-induced CTLA-4 and Foxp3 expression was better than anti-Mertk Ab (Figures 6(a)–6(d)).

**Figure 7:**
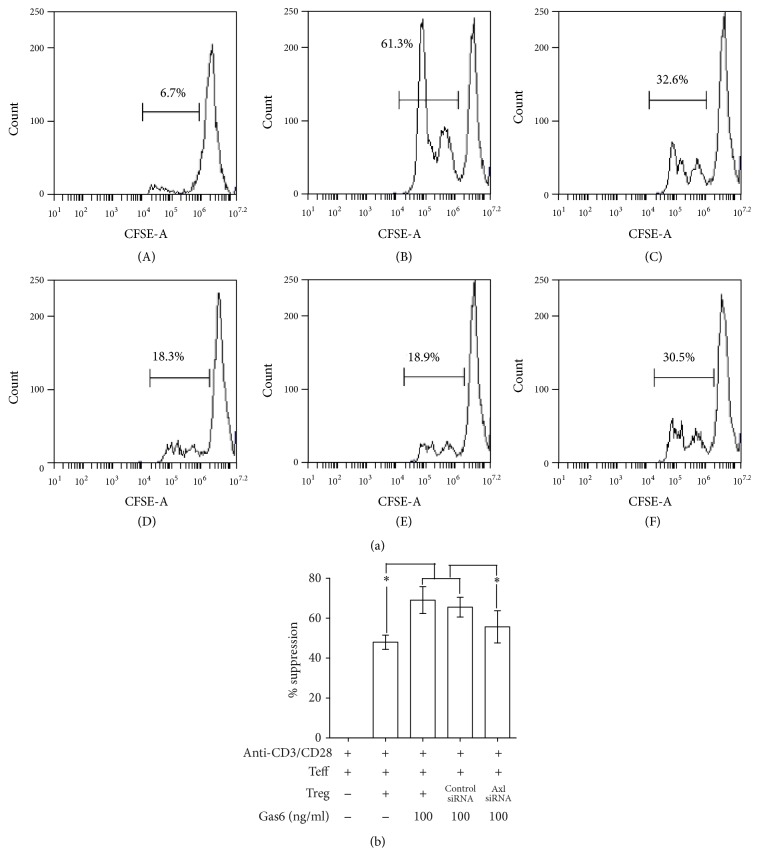
Gas6 modulates the suppressive function of Tregs mainly through Axl receptor. Axl knockdown Tregs were pretreated with 100 ng/ml rmGas6 for 24 h and then mixed with CD4^+^CD25^−^T cells in presence of anti-CD3/CD28 Abs. After 72 h, the suppressive function of CD4^+^CD25^+^T cells was determined by CFSE assay (a and b) (*n* = 4/group). (A) CD4^+^CD25^−^T cells alone. (B) CD4^+^CD25^−^T cells were stimulated with a combination of soluble anti-CD3 and anti-CD28 antibodies. (C) CD4^+^CD25^+^Tregs and (D) rmGas6-pretreated Tregs were mixed with CFSE-labeled CD4^+^CD25^−^T cells at a ratio of 1 : 1 in the presence of anti-CD3 and anti-CD28 antibodies. (E) siRNA-control and (F) siRNA-Axl transfected CD4^+^CD25^+^Tregs pretreated with Gas6 were cocultured with CFSE-labeled CD4^+^CD25^−^T cells. ^*∗*^*P* < 0.05.

The elevated expression of CTLA-4 and Foxp3 in Tregs after Gas6 stimulation was abrogated after Axl knockdown by siRNA (Fig. S4 A, Figures 6(e)–6(h)). No significant differences were found in Tregs viability between Axl-siRNA group and control group (Fig. S4B). Axl knockdown did not affect the level of TGF-*β*1 (Fig. S5A). Gas6-induced enhancement of suppressive activity of Tregs on T cell proliferation was abrogated after Axl knockdown as measured by CFSE assays (Figures 7(a) and 7(b)). Similarly, IL-2 levels were higher in Axl-siRNA group than control group (Fig. S5B).

## 4. Discussion

Growth arrest-specific 6 (Gas6) is a secreted vitamin K-dependent protein present in the human circulatory system [8, 9]. Using Gas6(−/−) mice, Gruber et al. found that Gas6 was critical for dampening the inflammatory during experiment autoimmune encephalomyelitis [22]. In an animal model of acute infection, a reduction of lung injury and cytokine production was observed after Gas6 administration [23]. Additionally, the inverse correlation between plasma Gas6 concentration and proinflammatory cytokines was observed in patients with type 2 diabetes [24]. In the current study, we found that systemic delivery of rmGas6 in mice increased the level of Foxp3 in CD4^+^CD25^+^Tregs. In addition, the CD4^+^CD25^+^Tregs from Gas6-administrated mice showed increased suppressive activity when compared with control mice. As Tregs play a crucial role in controlling inflammation, the anti-inflammatory effect of Gas6 may be, at least in part, mediated through increasing the suppressive function of Tregs.

The direct effect of Gas6 on the functions of Tregs was confirmed by in vitro study. Gas6 stimulation increased the expression of CTLA-4 and Foxp3 as well as the suppressive activity of Tregs. Tregs can attenuate IL-2 homeostasis by inhibiting IL-2 production and/or excessive IL-2 consumption [25]. We found that IL-2 levels in ex vivo culture supernatants were positively correlated the results of CFSE test. These results indicate that Gas6 enhances Tregs mediated suppression of CD4^+^T lymphocytes through increasing the ability of Tregs to consume IL-2 or suppressing IL-2 production. There were evidences that Tregs suppress Th1 and Th2 type cytokine secretion and contribute to Th2 polarization [26]. In the present study, we found that Tregs induced type 2-T cell polarization in vitro, but it was not affected by Gas6 stimulation. In contrast, a reduction of splenic T-helper 1 cell was observed in an animal model of rheumatoid arthritis after Gas6 overexpression [27], and the mechanism needs to be further investigated.

Gas6 is the ligand for the TAM family of receptors, which is composed of 3 members: Tyro3, Axl, and Mertk [8, 9]. In addition to its role in tissue homeostasis which is evident in the adult nervous, reproductive, and vascular systems, Gas6/TAM signaling also has especially profound effects in the regulation of innate immune response [10]. Mice that lack TAM receptors develop a severe lymphoproliferative disorder [14]. Because previous studies illustrated that TAM receptors are not expressed in most lymphocytes, the lymphoproliferative disorder in TAM receptors knockout mice had been attributed to hyperactivation of antigen-presenting cells [14–16]. In this study, we found that CD4^+^CD25^+^Tregs also expressed the TAM receptors Mertk and Axl. Blocking Axl receptor was more effective than blocking Mertk receptor at abrogating the Gas6-induced CTLA-4 and Foxp3 expression in CD4^+^CD25^+^Tregs. This result was confirmed by Axl knockdown which resulted in decreased expression of CTLA-4 and Foxp3 in Tregs as well as the suppressive activity of the cells after Gas6 stimulation. Our results showed the effect of Gas6 on suppressive activity of Tregs mainly through Axl receptor, which is supported by the report that Gas6 binds the TAM receptors with different affinities: Axl ≥ Tyro3 ≫ Mertk [28, 29].

## 5. Conclusion

The present study indicates that Gas6 has a direct effect on the functions of CD4^+^CD25^+^Tregs. Gas6 enhances the suppressive activity of Tregs mainly through Axl receptor. As the crucial role of Tregs in regulating ongoing immune responses and maintaining self-tolerance, our finding may be helpful in seeking for a novel interventional strategy against immune-related diseases, including sepsis, injury, and rheumatoid arthritis as well as diabetes.

## Supplementary Material

Figure S1: Protein levels of Axl and Mer in CD4+CD25− T cells.
Figure S2: Detection of TAM receptors on CD4+CD25+Tregs surface using a fluorescence microscopy.
Figure S3: Effects of Gas6 on TGF-β1 production in CD4+CD25+Tregs in vitro.
Figure S4: The transfection efficiency of lentiviral vectors for CD4+CD25+Tregs.
Figure S5: Effects of knockdown of Axl on TGF-β1 production by Tregs and IL-2 levels in co-culture supernatants.
