# Supplementary material for: Growth Arrest-Specific 6 Enhances the Suppressive Function of CD4+CD25+ Regulatory T Cells Mainly through Axl Receptor
Source: Mediators Inflamm. 2017 Feb 8;2017:6848430. doi: 10.1155/2017/6848430 (PMC5320320; doi:10.1155/2017/6848430)
Supplement: Supplementary file 1 — Figure S1: Protein levels of Axl and Mer in CD4+CD25− T cells. Figure S2: Detection of TAM receptors on CD4+CD25+Tregs surface using a fluorescence microscopy. Figure S3: Effects of Gas6 on TGF-β1 production in CD4+CD25+Tregs in vitro. Figure S4: The transfection efficiency of lentiviral vectors for CD4+CD25+Tregs. Figure S5: Effects of knockdown of Axl on TGF-β1 production by Tregs and IL-2 levels in co-culture supernatants. [file 6848430.f1.pdf]

Growth arrest-specific 6 enhances the suppressive function of CD4+CD25+ regulatory T cells mainly through Axl receptor

Guang-ju ZHAO,Jia-yi ZHENG, Jia-lan BIAN, Long-wang CHEN, Ning DONG,

Yan YU, Guang-liang Hong, Arvine Chandoo, Yong-ming YAO, Zhong-qiou LU

Supplementary Information

Supplemental Figures

Figure S1

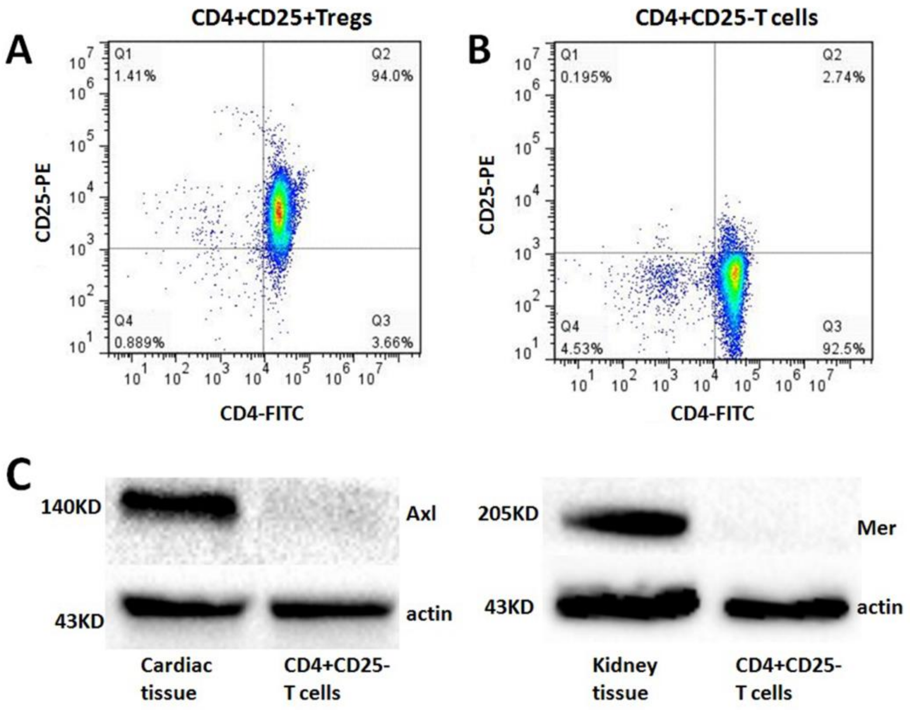

Figure S1. $CD4^+CD25^+$ Tregs and $CD4^+CD25^-$ T cells were isolated from the mice splenic cells in two steps by MACS system according to the manufacturer's instructions. The purity of $CD4^+CD25^+$Tregs (A) and $CD4^+CD25^-$ T cells (B) were analyzed using flow cytometry (FCM). C. protein levels of Axl and Mer in $CD4^+CD25^-$ T cells were analyzed by Western blot using specific anti-Axl and anti-Mer antibodies.

Figure S2

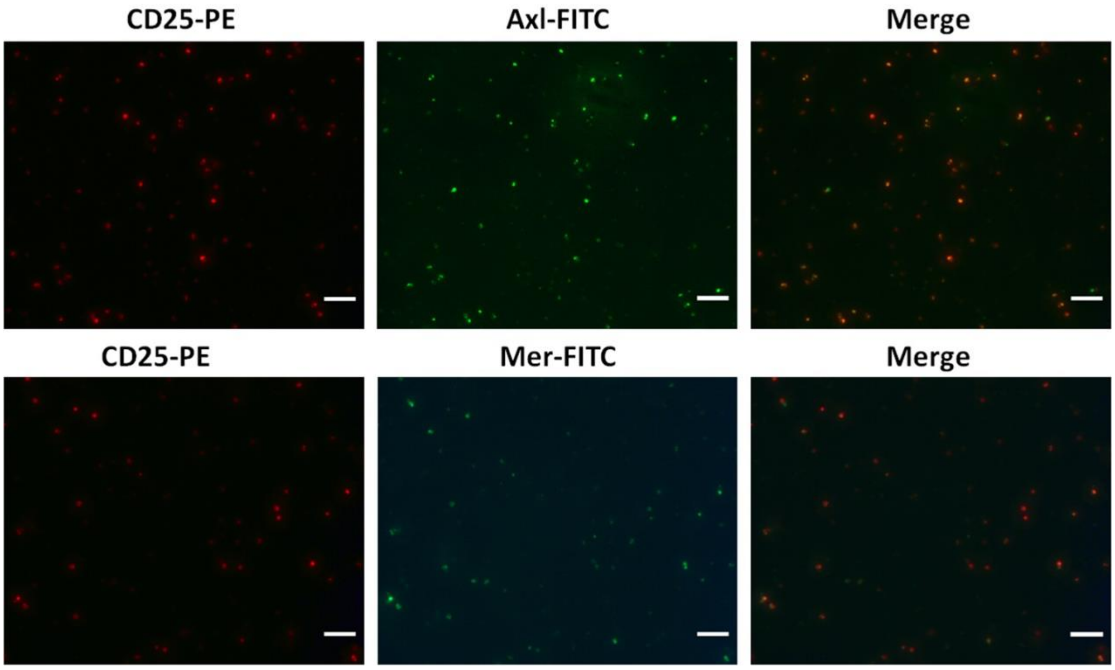

Figure S2. Detection of TAM receptors on CD4$^+$CD25$^+$Tregs surface using a fluorescence microscopy. CD4$^+$CD25$^+$Tregs stained with anti-Axl and anti-Mer monoclonal Ab, respectively, following with FITC-labelled Goat anti-Rat IgG as the secondary Ab. The cells were analyzed by a fluorescence microscopy. Representative photomicrographs show that FITC-positive cells (green) were detected among PE-positive CD4$^+$CD25$^+$Tregs (red). Those double-stained cells are shown in yellow.

Figure S3

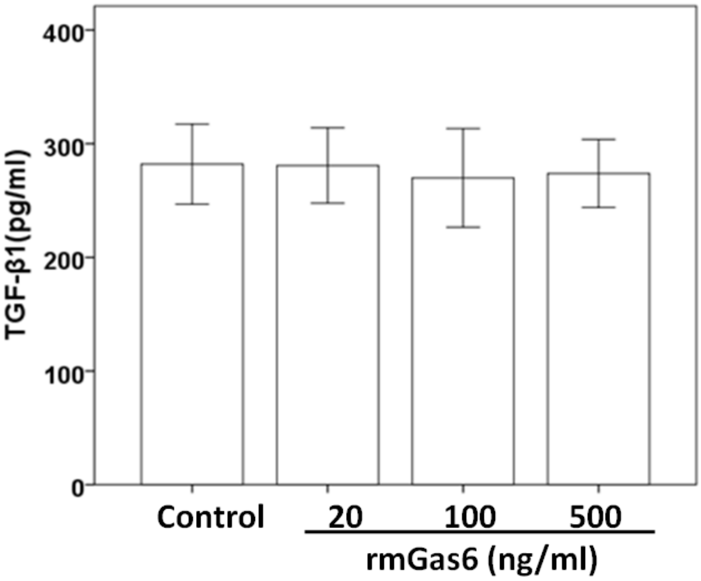

Figure S3. Effects of Gas6 on TGF-β1 production in CD4+CD25+Tregs in vitro. $CD4^+CD25^+$Tregs were treated with 20, 100 and 500ng/ml Gas6 for 24h, the production of TGF-β1 was determined by ELISA (n=4 in each group).

Figure S4

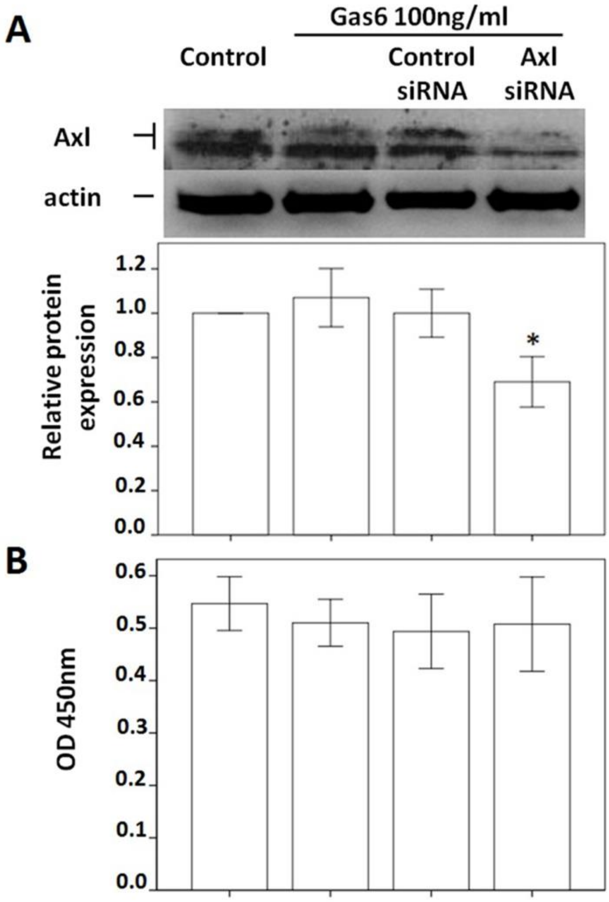

Figure S4. CD4⁺CD25⁺Tregs were transfected with LV-Axl-siRNA or LV-control-siRNA following 100ng/ml Gas6 stimulation. A. A typical western blot and average values, respectively, for Axl protein abundance in Tregs were illustrated (n=3/group). B. cell viability was measured at a wave length of 450nm using the CCK-8 assay (n=6/group). *, P<0.05 compared with the value for the Gas6 group.

Figure S5

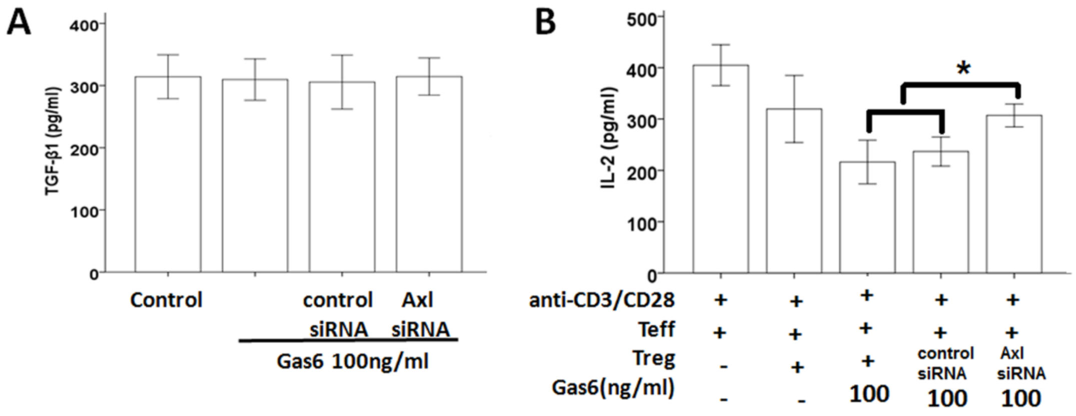

Figure S5. Effects of knockdown of Axl on TGF-β1 production by Tregs and IL-2 levels in co-culture supernatants. A. Axl knockdown Tregs were pretreated with 100ng/ml rmGas6 for 24h, and the production of TGF-β1 was determined by ELISA (n=4/group). B. siRNA-Axl or siRNA-control transfected $CD4^+CD25^+$Tregs pretreated with 100ng/ml rmGas6 were co-cultured with $CD4^+CD25^-$T cells. IL-2 levels in co-culture supernatants were measured by ELISA.*, $P<0.05$ compared with Axl-siRNA group.
